# Dual targeting of glutaminase 1 and thymidylate synthase elicits death synergistically in NSCLC

**DOI:** 10.1038/cddis.2016.404

**Published:** 2016-12-08

**Authors:** Jae-Seon Lee, Joon H Kang, Seon-Hyeong Lee, Dongwan Hong, Jaekyoung Son, Kyeong M Hong, Jaewhan Song, Soo-Youl Kim

**Affiliations:** 1 Cancer Cell and Molecular Biology Branch, Division of Cancer Biology, National Cancer Center, Goyang, Gyeonggi-do 410-769, Republic of Korea; 2 Department of Biochemistry, College of Life Science and Biotechnology, Yonsei University, Seoul 120-749, Republic of Korea; 3 Cancer Immunology Branch, Division of Cancer Biology, National Cancer Center, Goyang, Gyeonggi-do 410-769, Republic of Korea; 4 Department of Biomedical Sciences, University of Ulsan College of Medicine, Seoul 138-736, Republic of Korea; 5 Omics Core Laboratory, National Cancer Center, Goyang, Gyeonggi-do 410-769, Republic of Korea

## Abstract

Glutaminase 1 (GLS1) expression is increased in non-small cell lung cancer (NSCLC). GLS1 knockdown using siRNA or inhibition using bis-2-(5-phenylacetamido-1,3,4-thiadiazol-2-yl)ethyl sulfide (BPTES) induced cell cycle arrest with significant reduction of ATP level while levels of reactive oxygen species or glutathione were not affected in NSCLC cell lines. Recently we found that NSCLC significantly depends on cytosol NADH for ATP production. GLS1 remarkably contributes to ATP production through transferring cytosolic NADH into mitochondria via malate-aspartate shuttle by supply of glutamate in NSCLC. Regulation of malate-aspartate shuttle by knockdown or inhibition of glutamic-oxaloacetic transaminase 2 or malate dehydrogenase 2 mimicked GLS1 knockdown, which induced cell death with ATP reduction in NSCLC. Therefore, GLS1 inhibition induced cell cycle arrest with ATP depletion by glutamate reduction. Dual inhibition with BPTES and thymidylate synthase inhibitor, 5-fluorouracil (5-FU), elicits cell death synergistically through cell cycle arrest in NSCLC. A preclinical xenograft model of NSCLC showed remarkable anti-tumour effect synergistically in the BPTES and 5-FU dual therapy group.

Glutamine metabolism regulated by cancer-specific glutaminase (EC 3.5.1.2, glutaminase 1 (GLS1), L-glutaminase and glutamine aminohydrolase) has been gaining attention in cancer biology as it has been reported that high levels of kidney-type glutaminase (glutaminase 1, GLS1, KGA) are associated with oncogenic activation. *MYC*-induced tumours reportedly exhibit increased expression of GLS1.^1, 2, 3^ Furthermore, both GLS1 knockdown or inhibition using small molecules^4^ diminished tumorigenesis in a hepatocellular carcinoma xenograft model.^5^

Fast growing cells require glucose and glutamine for biosynthesis. However, cancer cell dependency on glutamine cannot be explained by nitrogen demand for nucleotide synthesis alone. Glutamine conversion into the TCA cycle intermediate *α*-ketoglutarate via glutamate,^2^ which is catalysed by GLS1 and glutamate dehydrogenase, is also essential for Kras-induced anchorage-independent growth.^6^ This suggests that the production of *α*-ketoglutarate (*α*-KG) catalysed by GLS1 and glutamate dehydrogenase is further catabolized to citrate, which turns into acetyl-CoA for fatty acid synthesis in glioblastoma cells.^7^ Glutamate has also been proposed to assist in the reduction of reactive oxygen species by glutathione synthesis through *γ*-glutamylcysteine.^1, 6^ Based on previous reports, glucose and glutamine are considered to support cancer cell anabolism instead of energy metabolism.

Recently, we found that GLS1 expression is highly upregulated in most non-small cell lung cancer (NSCLC) cell lines compared with that in normal. NSCLC cells significantly depended on cytosol NADH to produce ATP through NADH transportation system, malate-aspartate shuttle (MAS).^8^ This is concurred with a previous report that oxidative phosphorylation is the major ATP supplier regardless of the rate of glycolysis.^9^ Recent studies have revealed that the mitochondrial membrane potential of cancer cells is very active, which suggests that cancer cells undergo active oxidative phosphorylation even though TCA cycle is not active (reviewed in Moreno-Sanchez *et al*^10^). MAS requires glutamate to supply oxaloacetate from mitochondria to cytosol via conversion to aspartate. GLS1 inhibition or glutamine depletion in culture media reduced cell growth and ATP production significantly in NSCLC cell lines.^11^ Here we explored the mechanism of GLS1-dependent energy production in NSCLC and assessed the best combination of critical metabolic regulation with GLS1 inhibition that may reverse the growth of NSCLC.

## Results

### High expression of GLS1 inversely correlates with overall survival in NSCLC

**Figure 1:**
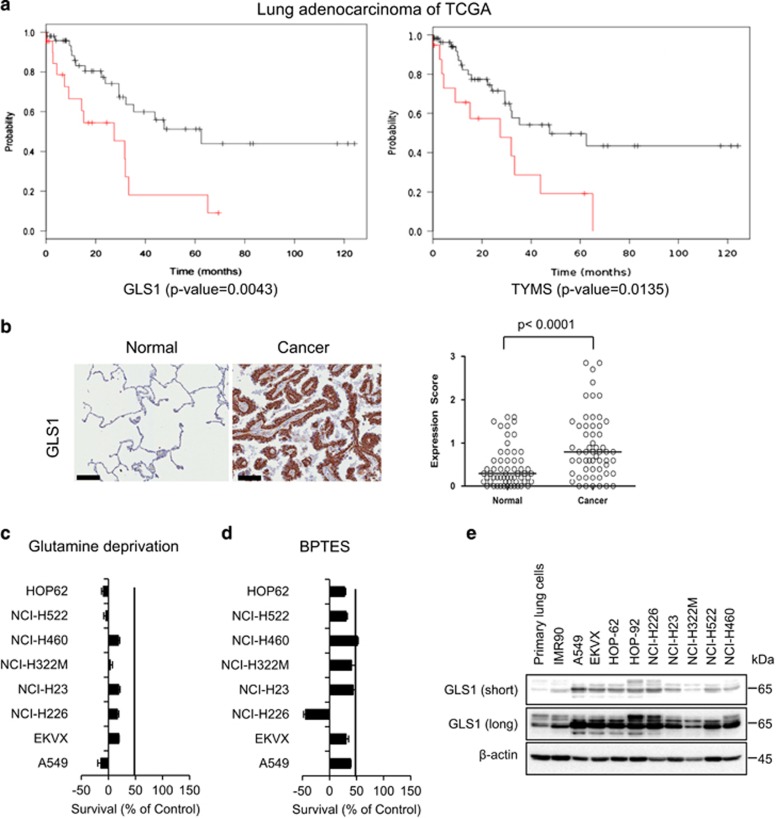
Glutamine metabolism is critical for overall survival and proliferation of NSCLC. (**a**) As was the case with the clinical information dataset from TCGA, higher levels of expression of GLS and TYMS were associated with reduced overall survival. Log-rank *P*-values of the genes were 0.0043 and 0.0135, respectively. Cases with higher expression levels of GLS and TYMS are coloured red, and cases without higher expression levels of these genes are coloured black. (**b**) Immunohistochemical staining of GLS1. The expression level of GLS1 in NSCLC tumour tissues was significantly higher than that in penumocytes from normal lung tissues (*N*=57).The expression score was obtained from immuno-staining intensity and the percentage of positive cells in tissue microarray core as in Materials and Methods. The expression score was significantly higher in NSCLC tumor cells than in normal lung pneumocytes (*P*<0.0001 by Mann-Whitney U test). (**c**) NSCLC cells were treated with glutamine-free medium for 48 h, and cell proliferation was tested by the SRB assay. (**d**) NSCLC cells were treated with GLS1 inhibitor, bis-2-(5-phenylacetamido-1,3,4-thiadiazol-2-yl)ethyl sulfide (BPTES, 100 μM), for 48 h, and cell proliferation was tested by the SRB assay. (**e**) The expression level of GLS1 in NSCLC cells and the primary small airway epithelial cells IMR90 was analysed by immunoblotting. SRB, sulforhodamine B

In order to perform a survival analysis of NSCLC, we gathered clinical information on lung adenocarcinoma patients from The Cancer Genome Atlas (TCGA).^12^ Kaplan–Meier analysis demonstrated that higher than average expression levels in GLS1 and thymidylate synthase (TYMS) gene expression were associated with poorer overall survival. In the TCGA datasets, 22.6 and 20.5% of cases (33/146 and 30/146) exclusively exhibited higher than average expression levels of GLS and TYMS, respectively (Figure 1a).

By the analysis of tissue microarray, the level of GLS1 expression in NSCLC (*N*=57) was significantly higher ((*P*<0.0001) by Chi-square test) than in type I or type II pneumocytes from normal lung tissues (*N*=57), which were obtained around tumour areas (Figure 1b and Supplementary Figure 1). In normal lung tissues, the expression of GLS1 was variable depending on the activated states of pneumocytes. Strong expression of GLS1 was observed in activated pneumocytes from highly inflammatory normal lung tissues, but the expression level was moderate to low in most normal pneumocytes (Supplementary Figure 1a). In contrast, the expression of GLS1 was strong or moderate in most NSCLC cases (Supplementary Figure 1b), and the cases showing low or negative expression of glutaminase in NSCLC tissues (Figure 1b) were less than those in normal lung tissues. We found that the A549 human lung adenocarcinoma cell line showed selective glutamine dependency for growth compared with the other cancer cells among the metabolic challenges (data not shown). Therefore, we tested whether NSCLC shows a common dependency on glutamine depletion or GLS1 inhibition using bis-2-(5-phenylacetamido-1,3,4-thiadiazol-2-yl)ethyl sulfide (BPTES). Interestingly, eight different NSCLC cell lines regardless of oncogenic differences showed universal dependency on glutamine as well as GLS1 inhibition using BPTES (Figures 1c and d). This result is consistent with the expression level of GLS1 by immunoblotting, which showed increased level of GLS1, compared with that in normal primary epithelial cells or immortalized fibroblast IMR90 (Figure 1e).

### GLS1 inhibition reduces ATP synthesis by glutamate depletion

**Figure 2:**
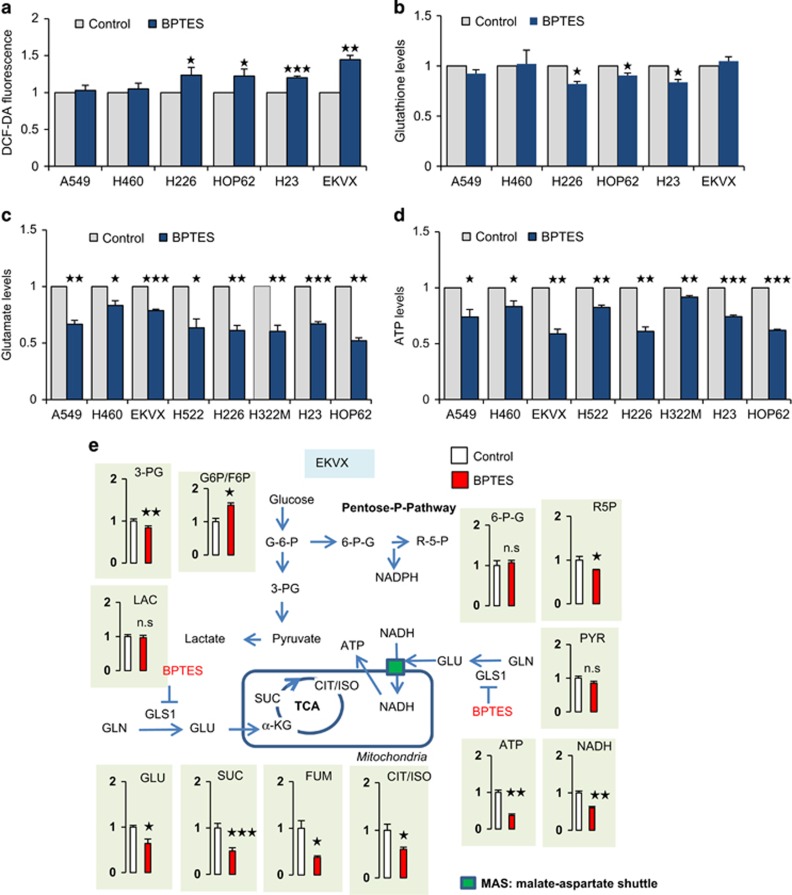

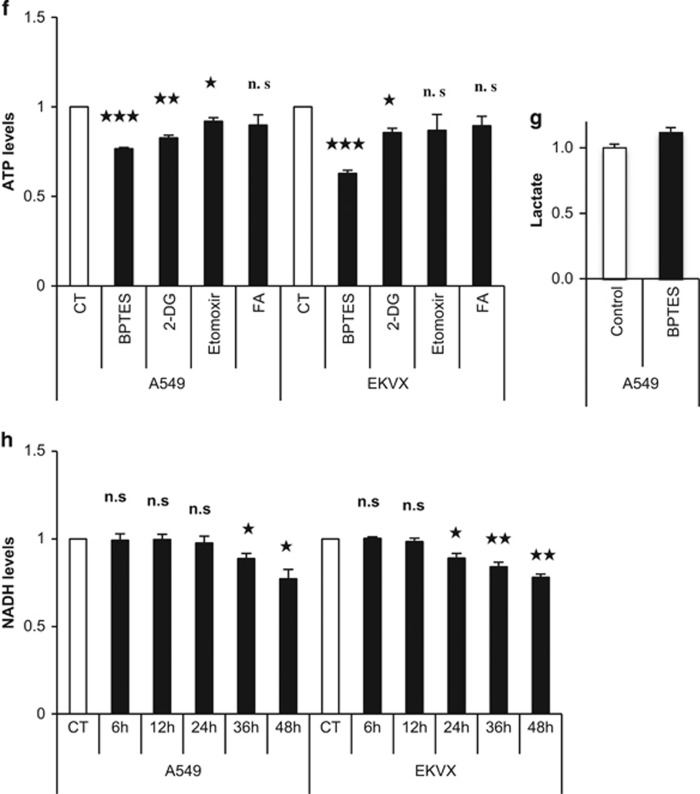
GLS1 inhibition generally correlates with ATP reduction. (**a**) Intracellular levels of ROS were measured in NSCLC with or without 10 μM BPTES treatment. (**b**) Intracellular levels of glutathione were measured in NSCLC with or without 10 μM BPTES treatment. (**c**) Glutamate levels were measured using a Glutamate Assay Kit after NSCLC cells were treated with 10 μM of BPTES for 48 h. (**d**) ATP levels were measured using an ATP Assay Kit after NSCLC cells were treated with 10 μM of BPTES for 48 h. (**e**) Relative pool sizes of metabolomics were assessed by targeted LC-MS/MS upon EKVX treated with 10 μM of BPTES for 48 h. Metabolite levels were measured in triplicate. (**f**) Cells were treated with the indicated inhibitors for 48 h and then the level of ATP was determined (BPTES: 10 μM, 2-DG: 2 mM, Etomoxir: 100 μM, FA: 500 μM). (**g**) A549 cells were treated with BPTES (10 μM) for 48 h, and the secreted lactate level was measured. (**h**) The level of total NADH was determined at the indicated hours after cells were treated with 10 μM BPTES. *P*-values were determined using two-tailed Student's *t*-tests (ns, not significant; *0.01<*P*<0.05; **0.001<*P*<0.01; ***P<0.001)

The increase of GLS1 is often discussed in relation to its important role of scavenging ROS through gluthathione production in cMyc-activated cancer cells. In accordance with the previous result, there was change in the levels of ROS and glutathione after GLS1 inhibition in NSCLC (Figures 2a, b, and Supplementary Figure 2a). To understand the metabolic contribution of GLS1 in NSCLC, we analysed the changes in the levels of metabolites after GLS1 inhibition in EKVX (Figure 2e). We found that BPTES effectively reduced the levels of the metabolites of the TCA cycle, whereas there were no changes in the levels of metabolites in glycolysis and the pentose phosphate pathway (Figure 2e). Interestingly, we found that GLS1 inhibition remarkably reduced ATP levels by >50% in EKVX cell line (Figure 2e). The decrease in the levels of ATP by GLS1 inhibition was observed in eight different NSCLC cells, and BPTES mediated a 20–50% decrease in glutamate, which correlated with a 10–50% decrease in ATP production (Figures 2c and d). In Figure 2e, ATP levels reduced by GLS1 inhibition showed an association with NADH but not with glycolysis. We have tested ATP levels with treatment of 2-DG blocking glycolysis, fluoroacetate blocking TCA via aconitase, etomoxir blocking fatty acid oxidation and BPTES blocking GLS1 separately (Figure 2f). Cells were treated with the indicated inhibitors for 48 h, and the level of ATP was determined. Reduction of ATP production was about 30% of control by BPTES treatment while other inhibitions reduced about 10% of control ATP production in A549 and EKVX. This suggests that the major energy source of NSCLC depends on glutamine instead of glucose or fatty acids. We have tested whether BPTES treatment may induce the excretion of lactate using A549 and EKVX. A549 cells were treated with BPTES (10 μM) for 48 h and secreted lactate was measured. The secretion was increased about 5% in A549 cells (Figure 2g) but it was not significant compared to the reduction of glutamate (Figure 2c). This suggests that NSCLC requires glutamine for energy production, which meant that glucose is not considered as an alternative energy source when the glutamate supply is blocked in NSCLC.

Further tests were conducted on whether cancer cells change GLS1 dependency for ATP production under hypoxia. Cells were treated with BPTES for 48 h, and the level of ATP was determined under normoxia and hypoxia conditions. Hypoxia reduced ATP production about 10% both in A549 and EKVX (Supplementary Figure 2b). BPTES treatment reduced about 30% ATP production under normoxia, and an additional 10% reduction of ATP production was observed under hypoxia in EKVX (Supplementary Figure 2b). This suggests that GLS1 dependency for ATP production was not changed by the hypoxia condition. We have also tested whether the glycerol 3-phosphate shuttle may contribute to the NADH transportation into mitochondria (Supplementary Figure 2c). However, we could not detect any significant change after the glycerol 3-phosphate dehydrogenase inhibitor treatment (Supplementary Figure 2c).

MAS is a shuttle, and nothing is consumed or generated by the MAS net reaction except for the transfer of NADH into mitochondria, with the total NADH amount being the same. We have tested whether the NADH amount should be the same when we inhibit GLS1 because MAS transfers NADH from cytosol to mitochondria (Figure 2h). To test this hypothesis, we have measured NADH levels at various time points including 0, 6, 12, 24, 36, 48 h. In the early time period after BPTES treatment, the total amount of NADH was not changed until 24 h in EKVX and 36 h in A549 (Figure 2h). However, the total amount of NADH was reduced in a time-dependent manner, suggesting less transportation of NADH by MAS inhibition results in less ATP production, which slows down anabolism in the longer period of BPTES treatment. Therefore, BPTES treatment finally may result in less production of NADH.

### GLS1 contributes ATP production through transferring cytosol NADH to mitochondria via malate-aspartate shuttle (MAS)

**Figure 3:**
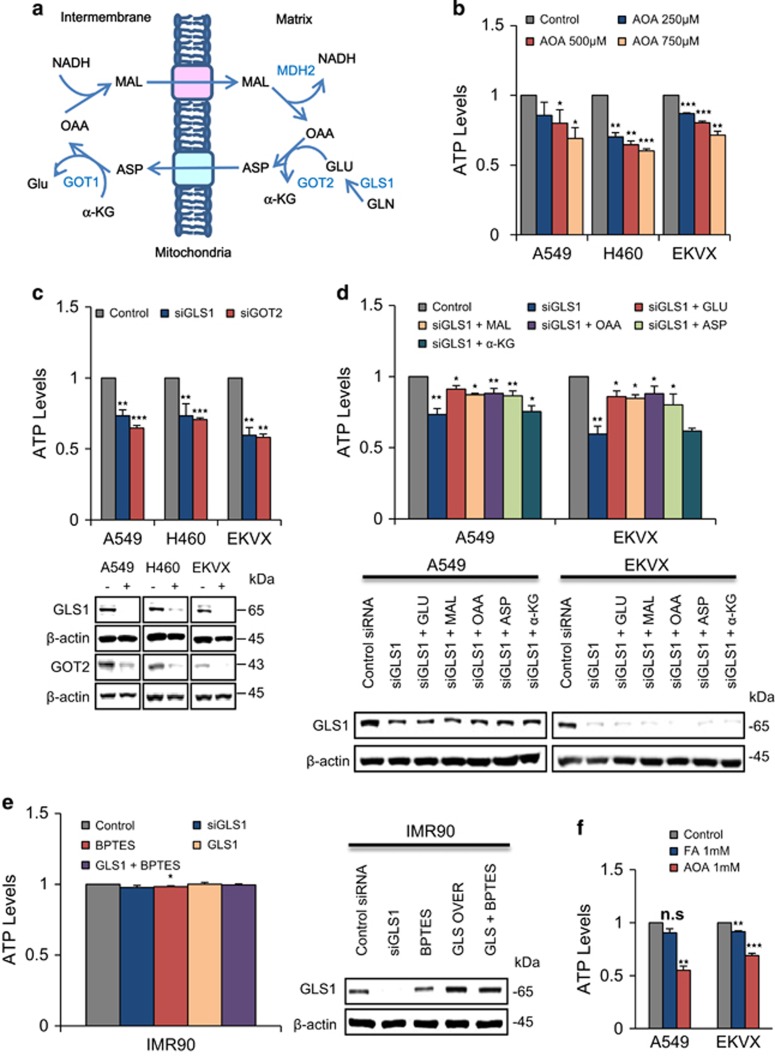
Blocking malate-shuttle system mimics GLS1 inhibition in ATP reduction. (**a**) A simplified model of the malate–aspartate shuttle (MAS) for NADH transportation into the mitochondrial matrix. (**b**) ATP levels were measured after treatment with 250 μM, 500 μM and 750 μM of AOA for inhibition of GOT2 in A549, H460 and EKVX for 48 h in a dose-dependent manner. (**c**) ATP levels were measured after treatment with 20 nM of siRNAs of GLS1 and GOT2 in A549, H460 and EKVX for 48 h. (**d**) ATP levels were measured after NSCLC cells were treated with 20 nM siRNA of GLS1 for 24 h and supplemented with metabolites, including 5 mM of malate, aspartate, glutamate and oxaloacetate, and 2 mM of dimethylated *α*-ketoglutarate, for 48 h. (**e**) ATP levels were measured using an ATP Assay Kit after treatment of IMR90 cells with 10 μM of BPTES for 48 h as a normal control. (**f**) ATP levels were measured after treatment with 1 mM fluoroacetate and 1 mM aminooxyacetate in A549 and EKVX for 48 h. *P*-values were determined using two-tailed Student's *t*-tests (ns, not significant; *0.01<*P*<0.05; **0.001<*P*<0.01; ***P<0.001)

Recently we found that cytosol NADH production contributed to significant part of ATP production through oxidative phosphorylation via MAS transportation in NSCLC. Of the key mechanisms of ATP production, there are three main pathways: direct glycolysis, NADH production via TCA cycle inside mitochondria, and transfer of cytosolic NADH produced by various dehydrogenases into mitochondria through the malate-aspartate shuttle (MAS) system (Figure 3a). To test whether MAS may be a major contributor of ATP production through transferring cytosolic NADH into the mitochondria in NSCLC, we inhibited or knocked down MAS (Figures 3b and c). Aminooxyacetic acid (AOA) inhibits glutamic oxaloacetic transaminase 1/2 (GOT1/2), a major participant in MAS that requires glutamate. AOA treatment reduced ATP by up to 40% compared with the control in a dose-dependent manner in A549, H460 and HOP62 (Figure 3b). GLS1 knockdown reduced glutamate levels, and ATP production decreased by up to 40% furthermore, GOT2 knockdown also resulted in approximately 40% reduction in ATP production (Figure 3c), which suggests that NSCLC depends on the MAS system for ATP supply through NADH transportation. We found that GLS1 knockdown critically reduced ATP levels, which can be recovered by glutamate, malate, aspartate or oxaloacetate supplement but not by dimethylated-*α*-KG (Figure 3d). GLS1 knockdown reduced ATP levels to 60% compared with the control, which was recovered to 90% by glutamate, malate and oxaloacetate supply in A549 and EKVX (Figure 3d). Interestingly, GLS1 expression did not affect ATP levels in the immortalized normal IMR90 cell line (Figure 3e). However, ATP levels were decreased about 50 and 40% in A549 and EKVX, respectively, after treatment with AOA for 48 h while ATP levels were less than 10% decreased by fluoroacetate treatment (TCA cycle blocker) (Figure 3f). This suggests that normal cells may obtain ATP from TCA cycle-mediated NADH while NSCLC obtains ATP from cytosolic NADH through the MAS system using glutamate.

### Glutamine oxidation and transaminase pathway were linked in MAS system

**Figure 4:**
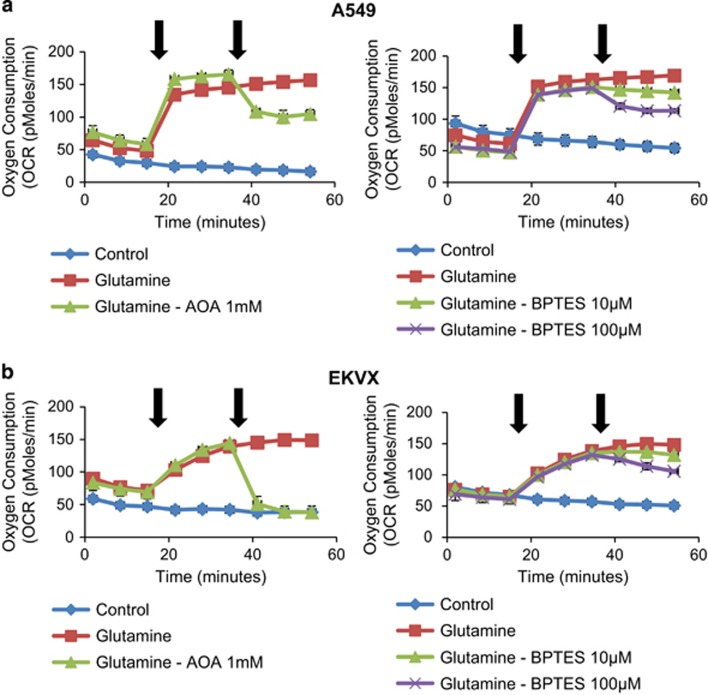
Glutamine oxidation and transaminase pathway is linked in the MAS system. (**a**) OCR response (% of baseline) in A549 cells to glutamine (4 mM), AOA (0 or 1 mM) and BPTES (0, 10 or 100 μM). (**b**) OCR response (% of baseline) in EKVX cells to glutamine (4 mM), AOA (0 or 1 mM) and BPTES (0, 10 or 100 μM). The % OCR was plotted using measurement 3 as the baseline. The assay medium was the substrate-free base medium. Each data point represents mean±S.D., *n*=3. Front arrow indicates addition of glutamine, and the second arrow indicates treatment of inhibitors

To test whether GLS1 is responsible for the supply of glutamate in the MAS system (Figure 3a), we have tested whether GLS1 inhibition using BPTES causes downregulation of oxygen consumption. Further, using the transaminase (GOT) inhibitor AOA, we tested whether transaminase is responsible for oxygen consumption. Oxygen consumption was measured using A549 and EKVX under three conditions including without glutamine, with glutamine, and with glutamine and AOA or BPTES. As shown in Figure 4, the addition of 4 mM glutamine to A549 and EKVX cells caused a threefold increase in oxygen consumption rate (OCR) (from 50 pmoles/min to 150 pmoles/min) revealing a high rate of oxygen consumption (Figure 4a and b). The addition of AOA following glutamine to A549 and EKVX cells abolished glutamine-induced OCR to 100 and 50 pmoles/min in A549 (Figure 4a) and EKVX (Figure 4b), respectively. Using BPTES, we tested whether GLS1 is also responsible for oxygen consumption via glutamate supply. The addition of BPTES following glutamine to A549 and EKVX cells decreased glutamine-induced OCR in a dose-dependent manner (Figures 4a and b). These suggest that transaminase-catalysed conversions of *α*-KG and aspartate is the major pathway for oxygen consumption, which is linked with the MAS system. GLS1 is the major pathway of glutamate supply in the MAS system.

### Dual treatment with BPTES and 5-FU significantly induces cell death associated with cell cycle arrest in NSCLC

**Figure 5:**
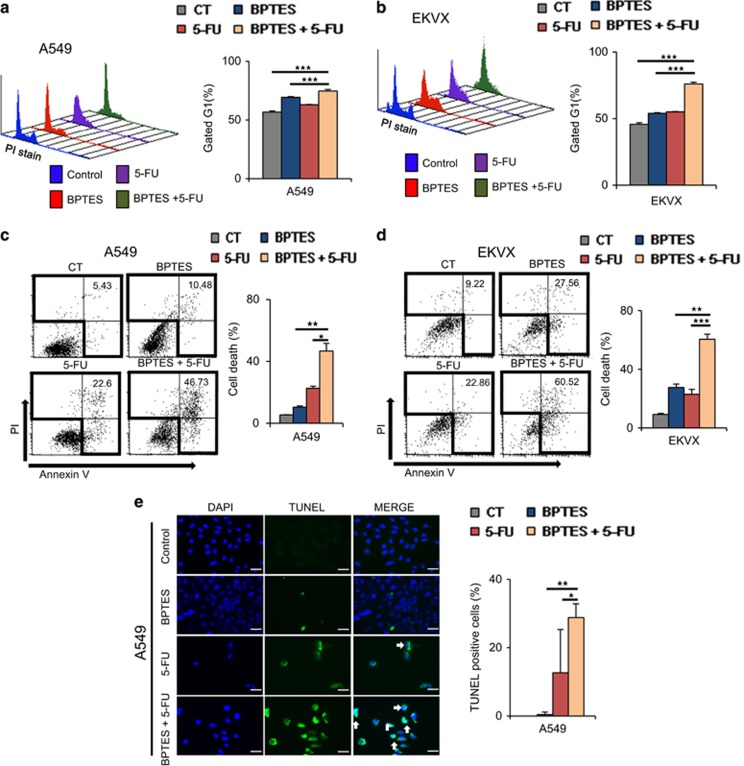

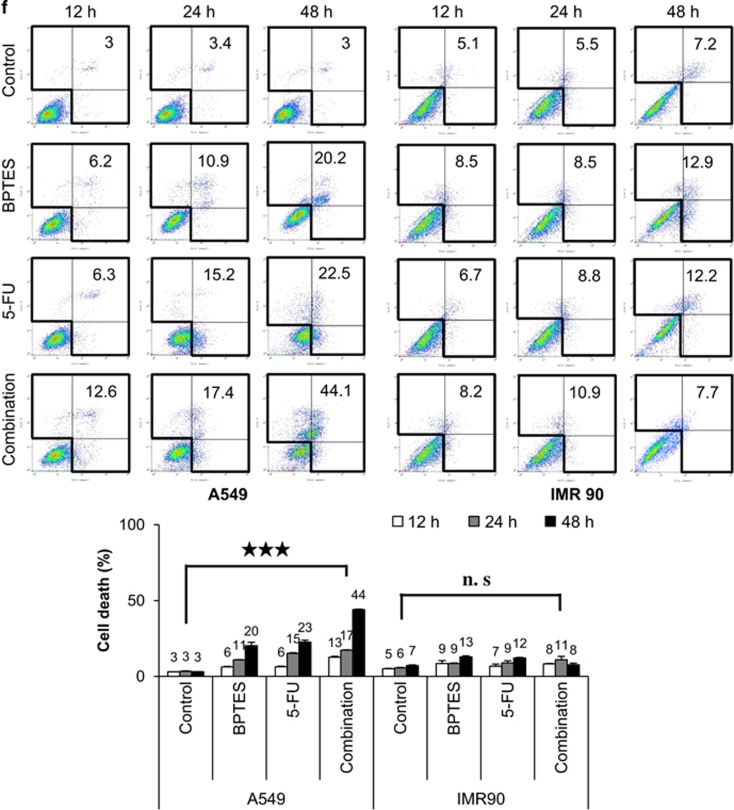
Cell death was significantly induced by dual treatment with BPTES and 5-FU through cell cycle arrest. To test the effect of combined BPTES and 5-FU treatment, NSCLC cells were treated with 10 μM of BPTES, 10 μM of 5-FU, or both 10 μM of BPTES and 10 μM of 5-FU for 48 h. (**a**) Cell cycle was measured by FACS analysis using PI staining after GLS1 inhibition with 10 μM of BPTES and/or 10 μM of 5-FU for 12 h in A549 cells. (**b**) Cell cycle was measured by FACS analysis using PI staining after GLS1 knockdown with 10 μM of BPTES and/or 10 μM of 5-FU for 12 h in EKVX cells. (**c**) Cell death was measured with FACS analysis using PI and annexin V in A549 after drug treatment for 48 h. (**d**) Cell death was measured with FACS analysis using PI and annexin V in EKVX after drug treatment for 48 h. (**e**) Cell death was measured with TUNEL staining in A549 after drug treatment for 48 h. The scale bar, 50 μm. (**f**) Cell death was measured by the annexin V assay after cells were treated with 10 μM of BPTES, 10 μM of 5-FU, or combined for 12, 24 and 48 h in A549 and IMR90. *P*-values were determined using two-tailed Student's *t*-tests (ns, not significant; *0.01<*P*<0.05; **0.001<*P*<0.01; ***P<0.001)

To test whether this synergistic effect is due to cell cycle arrest, we performed fluorescence-activated cell sorting (FACS) analysis using propidium iodide (PI) staining (Figures 5a and b). Inhibition by BPTES or 5-FU alone induced G1 cell cycle arrest at a rate of approximately10% in both A549 (Figure 5a) and EKVX (Figure 5b). Furthermore, dual BPTES and 5-FU treatment increased G1 cell cycle arrest to approximately 20–30% in A549 (Figure 5a) and EKVX (Figure 5b). The combined BPTES and 5-FU treatment clearly showed the synergistic effect of cell death by FACS analysis using PI and annexin V staining (Figures 5c and d). The combined BPTES and 5-FU treatment induced approximately 46–60% cell death, whereas BPTES alone induced approximately 10–27% cell death and 5-FU alone induced approximately 22% cell death in A549 (Figure 5c) and EKVX (Figure 5d) cells. This synergistic induction of cell death was observed in most of the NSCLC cell lines, including CALU-1, HOP62, H460 and H226, with approximately 42–71% cell death induction by the combined BPTES and 5-FU treatment (Supplementary Figure 3a). To measure the effect on apoptosis, BPTES treatment, 5-FU treatment, and combined BPTES and 5-FU treatment were analysed by terminal deoxynucleotidyl transferase dUTP nick-end labelling (TUNEL) assay (Figure 5e and Supplementary Figure 3b). The combined BPTES and 5-FU treatment showed approximately 28 and 31% positive TUNEL staining of fragmented DNA, respectively, whereas treatment with BPTES alone showed approximately 0.4 and 12% positivity in A549 (Figure 5e) and EKVX (Supplementary Figure 5b) cell lines, respectively. We have tested whether ATP depletion by BPTES, 5-FU, and combination of BPTES and 5-FU induces cell death following cell cycle arrest. Cell death was measured by the annexin V assay after cells using A549 and IMR90 were treated with BPTES, 5-FU, or combined both for 12, 24 and 48 h. After 48 h treatment, ATP depletion increased cell death about sevenfold, eightfold and 15-fold by BPTES, 5-FU and combination, respectively, in A549 cells while normal control cell IMR90 showed about less than 1.7-fold increase in all groups (Figure 5f).

### MAS knockdown combined with thymidylate synthase inhibition potentiates cell death induction in NSCLC

**Figure 6:**
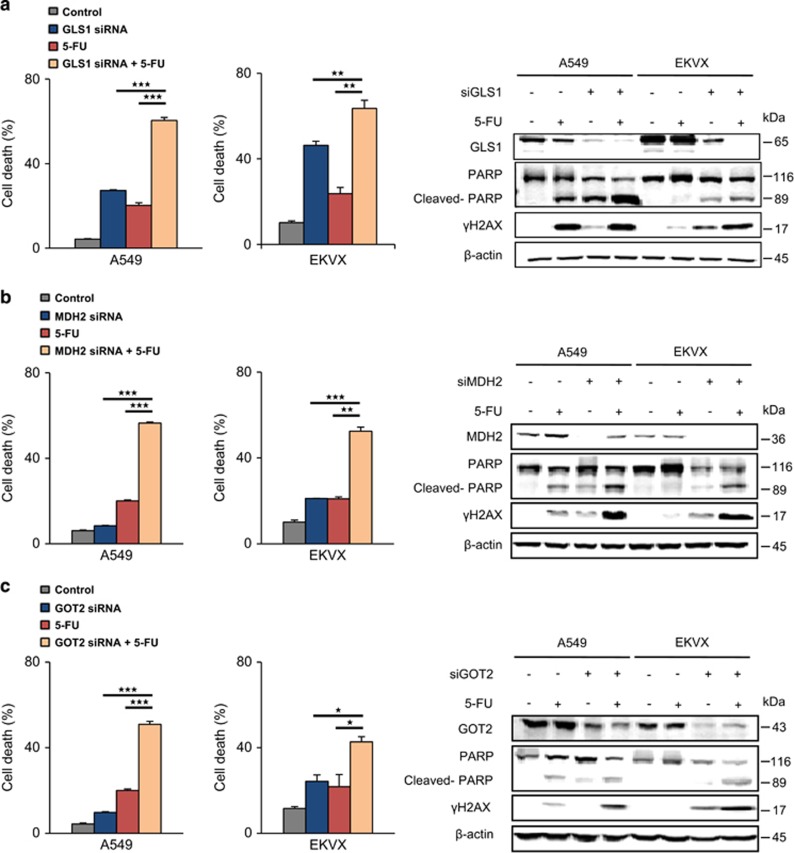
Cell death was significantly induced by 5-FU treatment with knockdown of MAS genes. (**a**) Cell death was analysed by FACS using PI and annexin V. Cells were treated 5-FU for 48 h after treatment with 20 nM siRNA of GLS1 for 24 h (Supplementary Figure 4a). GLS1 knockdown, *γ*H2AX and PARP cleavage was determined by immunoblotting. (**b**) Cell death was analysed by FACS using PI and annexin V. Cells were treated 5-FU for 48 h after treatment with 20 nM siRNA of MDH2 for 24 h (Supplementary Figure 4b). MDH2 knockdown, *γ*H2AX and PARP cleavage was determined by immunoblotting. (**c**) Cell death was analysed by FACS using PI and annexin V. Cells were treated 5-FU for 48 h after treatment with 20 nM siRNA of GOT2 for 24 h (Supplementary Figure 4c). GOT2 knockdown, *γ*H2AX and PARP cleavage was determined by immunoblotting. *P*-values were determined using two-tailed Student's *t*-tests (ns, not significant; *0.01<*P*<0.05; **0.001<*P*<0.01; ***P<0.001)

To test whether this synergistic effect is based on the failure of the MAS system due to glutamate depletion, cell survival was measured by FACS after GLS1, MDH2 and GOT2 were knocked down, respectively, in A549 and EKVX with or without 5-FU treatment (Figure 6 and Supplementary Figure 4). GLS1 combined with 5-FU knockdown induced up to 70% cell death, whereas knockdown of GLS1 alone showed 30–40% cell death induction by FACS analysis (Figure 6a and Supplementary Figure 4a). MDH2 or GOT2 knockdown with 5-FU treatment induced approximately 50% cell death, whereas knockdown of MDH2 (Figure 6b and Supplementary Figure 4b) or GOT2 (Figure 6c and Supplementary Figure 4c) alone showed approximately 10–20% cell death induction; moreover, 5-FU treatment resulted in approximately 20% cell death induction in A549 and EKVX cell lines. The combined therapeutic approach using 5-FU and MAS system inhibition using siRNAs of GLS1, MDH2 and GOT2 showed evident increase of cell death in PARP cleavage (Figures 6a–c). This suggests that TYMS inhibition by 5-FU has a synergistic effect with ATP depletion through MAS system inhibition.

### Dual treatment of BPTES and 5-FU reverses the A549 NSCLC xenograft model

**Figure 7:**
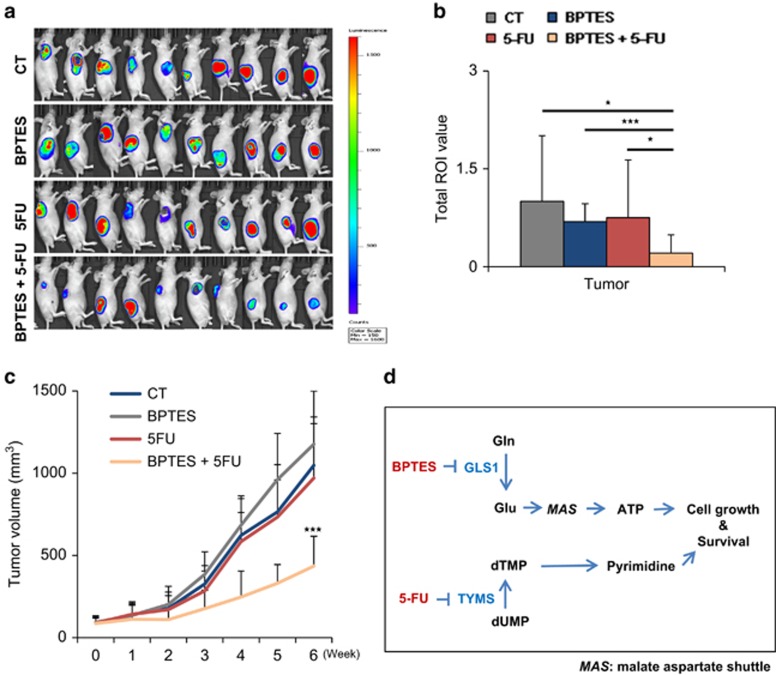
A preclinical NSCLC mouse model was reversed by dual treatment with BPTES and 5-FU. (**a**) A549-luciferase (5 × 10^6^) cells were injected in both flanks of 6–8-week-old BALB/c nude mice. When the volume of the tumour mass reached 80 mm^3^, the mice were randomly assigned to one of four treatment groups including vehicle control, BPTES, 5-FU and combination of BPTES and 5-FU (*n*=10 per group). BPTES (10 mg/kg body weight), 5-FU (20 mg/kg body weight), and vehicle were administered orally 5 days/week, and the tumour growth was monitored by photon flux released from luciferin as described in the experimental procedures. The data were expressed as photon flux (photons/s/cm^2^/steradian), which is represented by a colour scale. (**b**) Graph shows the total ROI value. (**c**) Graph represents the tumour growth curve as measured using calipers. (**d**) A proposed model of the synergistic mechanism between GLS1 inhibition and TYMS inhibition in NSCLC. *P*-values were determined using two-tailed Student's *t*-tests (ns, not significant; *0.01<*P*<0.05; *P*<0.01; ***P<0.001)

To test whether BPTES treatment has an anti-tumour effect when combined with 5-FU in a preclinical xenograft model of NSCLC, four groups were subjected to different treatments, including control, either BPTES or 5-FU treatment, and BPTES and 5-FU dual treatment. Interestingly, none of the single treatments showed any improvement; only the dual treatment showed a significant reduction in tumour growth (Figures 7a–c). Luciferase-tagged A549 cells were used for non-invasive measurement of the anticancer activity of BPTES and 5-FU. The tumour volume was measured using a Xenogen instrument with luciferin injection before killing the mice. The Xenogen images showed that the tumour size in the dual treatment group was one-third of that in the control group (Figures 7a and b). The tumour volume was also measured using calipers once a week to minimize the possibility of measurement errors (Figure 7c). After 42 days of treatment, the tumour volume of the combined treatment group was also reduced to about one-third compared with that in the control group (Figure 7c).

In summary, we propose that GLS1 inhibitor, BPTES, plus TYMS inhibitor, 5-FU, elicits death synergistically through cell cycle arrest in NSCLC (Figure 7d).

## Discussion

GLS1 levels increase substantially in NSCLC (Figure 1b), and this increase is inversely correlated with the overall survival rate in NSCLC (Figure 1a). Here we found that GLS1 knockdown or inhibition using BPTES induces cell death through G1 cell cycle arrest (Figures 5a and b), which potentiates an anticancer effect when it is combined with TYMS inhibition using 5-FU in NSCLC. Dual treatment with BPTES and 5-FU resulted in the reversal of NSCLC in a preclinical xenograft model (Figure 7a–c). Recently we have reported that BPTES treatment exacerbated pyrimidine supply through reduction of carbamoyl-phosphate synthesis, which induced cell death synergistically with 5-FU treatment in NSCLC.^11^ The link between GLS1 inhibition and TYMS inhibition remained to be explained. We may explain how the inhibition of glutamine metabolism is linked to the activity of TYMS in two directions. First, GLS1 knockdown or inhibition induces significant decrease of ATP production. ATP is one of the major components of nucleotide synthesis as a building material. Therefore we can understand that cell growth is naturally reduced due to lack of ATP supply. The second, there are previous reports on cell cycle progression delay in G2/M transition due to decrease of ATP level because there is ATP-dependent cell cycle checkpoints at G2/M transition.^13, 14^

The increased levels of TCA cycle intermediates supplied by GLS1 and glutamate dehydrogenase can be used as biosynthetic precursors in cancer.^2, 7^ The rapid turnover of glutamate to aspartate leads to the synthesis of nucleotides, asparagine and arginine.^7^ The Dang group demonstrated that *GLS1* knockdown or inhibition has benefits on the reduction of cancer growth in *Myc*-driven tumours such as hepatocellular carcinoma and B cell lymphoma.^5^ *Myc* regulates gene expression either directly, such as via glycolytic genes including lactate dehydrogenase A, or indirectly, such as via repression of the microRNA miR-23a/b to increase GLS1 expression.^1, 15^ Glutamine may participate in an alternative glucose-independent TCA cycle for producing energy only under conditions of hypoxia or glucose deprivation.^2^ Under high glucose conditions, however, glutamine carbons are not oxidized via the TCA cycle and produce only five ATP/mol glutamine converted to lactate plus CO_2_.^2^ This suggests that the activated glutaminolysis supports a significant proportion of the biosynthetic needs of the cells under aerobic conditions.

Interestingly, GLS1 knockdown or inhibition using BPTES reduced ATP levels (Figure 2c), which is in accordance with the results from previous reports.^1, 2^ This result then raises the question of how glutamate is linked to ATP synthesis under aerobic and hyper-glucose conditions. Glutamate is the primary nitrogen donor for the synthesis of non-essential amino acids through the production of *α*-KG, which participates in anaplerotic reactions.^16^ However, glutamate is also linked with physiologically essential energy metabolism known as an electron transfer system (MAS).^17^ MAS is a biochemical system for transferring NADH produced in the cytosol across the inner membrane of mitochondria for oxidative phosphorylation. The MAS biochemical system is required because the mitochondrial inner membrane is impermeable to NADH. The electrons of NADH enter the electron transport chain of the mitochondria via malate to generate ATP. In the MAS system, malate carries the electrons from NADH across the membrane. Here we found that oxidative phosphorylation through MAS is a major contributor of ATP production through NADH transportation in NSCLC, which is proved by the observation that GOT2 knockdown or AOA treatment resulted in almost the same effect as GLS1 knockdown (Figures 3b and c). MAS inhibition by blocking the glutamate supply with BPTES alone was not sufficient to induce critical cell death. However, dual BPTES and 5-FU treatment demonstrated great synergistic effect in cell death induction.

The toxicity of GLS1 inhibition may be explained by the reduction of glutathione in several aspects. Recently, it was reported that the combination of GLS1 and heat shock protein 90 (HSP90) inhibitors selectively triggered the death of cells presenting high mTORC1-mediated translation rates by TSC1/2 deficiency.^18^ GLS1 inhibition sensitized the death of cells with HSP90 inhibition through increased oxidative stress by depleting antioxidant glutathione. However, this conclusion was drawn under circumstances of over-activated mTORC1 and proteotoxic stress due to HSP90 inhibition. BPTES treatment can amplify ROS stress enough to induce cell death. BPTES treatment alone did not induce cell death in TSC1/2 deficiency, although BPTES treatment increased the ROS levels.^18^ Here we found that BPTES treatment alone showed on average <20% increase of ROS levels in NSCLC under normal growth conditions (Figure 2a). This suggests that GLS1 does not contribute much to glutathione production or to ROS consumption, which is inconsistent with the findings from previous reports.^1, 6^

As a proof of concept, we tested the effect of BPTES-induced GLS1 inhibition combined with 5-FU-induced TYMS inhibition and showed that NSCLC cell growth was abrogated and that cell death was induced with significant reduction of tumour growth (Figure 7). The favourable findings on the effect of this combined treatment may prove to be useful as a therapeutic approach to NSCLC.

## Materials and methods

### Overall survival analysis using TCGA data

Gene expression data from RNA-Seq of lung adenocarcinoma cancer patients at TCGA were collected. Gene expression data and clinical information were downloaded from TCGA data portal site (https://tcga-data.nci.nih.gov/tcga/). The parameters of data matrix of TCGA data portal are as follows: Select a disease: LUAD, Data Type: ‘Clinical' and ‘Expression-Genes', Batch Number: All, Data Level: Level3 and Availability: Available. After then, paired sequencing data consisting of matched cancer and normal tissues were selected using a custom-made script. In order to assess survival, we performed Kaplan–Meier analysis of GLS and TYMS gene expression in lung cancer patients using R software.

### Cell culture

NSCLC cell lines were obtained from the US National Cancer Institute (Bethesda, MD, USA) (MTA 1-2702-09). All cells were incubated at 37 °C and maintained at 5% CO_2_. H23, H226, IMR90 (normal lung fibroblast, ATCC CCL-186) and Lung Primary (Primary Small Airway Epithelial Cells; Normal, Human, ATCC PCS-301-010) cell lines were obtained from ATCC. IMR-90 cell was grown in DMEM/HIGH GLUCOSE medium (SH30243.01, Hyclone, Logan, UT, USA) containing 10% FBS. Lung primary cell was airway epithelial cell basal medium (PCS-300-030, ATCC, Manassas, VA, USA) with the bronchial epithelial cell growth kit (PCS-300-040, ATCC, Manassas, VA, USA).

NSCLC cells were grown in RPMI 1640 medium (Hyclone, Logan, UT, USA) plus 10% fetal bovine serum (FBS; Hyclone), penicillin and streptomycin. A small interfering RNA (siRNA) duplex targeting human GLS1, GOT2 and MDH2 (Santa Cruz, CA, USA) was introduced into the cells using Lipofectamine 3000 (Invitrogen, Carlsbad, CA, USA) according to the manufacturer's instructions. As negative controls, cells were incubated with Lipofectamine 3000 (Invitrogen) and a negative siRNA (Santa Cruz). The hypoxic condition was achieved by incubating the cells in 1% of O_2_, 94% of N_2_ and 5% of CO_2_ in a multigas incubator (Vision scientific. VS-9000GC, Seoul, Korea).

### Immunohistochemical staining of glutaminase

Tissue arrays (CC5, various human lung cancer tissues; CCN5, normal human lung tissues; *n*=57 each case) were purchased from SuperBioChip (Seoul, Korea). Immunohistochemical staining was performed using the UltravisionLP Detection System (Thermo Fisher Scientific Inc., Fremont, CA, USA). Briefly, after deparaffinisation of formalin-fixed, paraffin-embedded human NSCLC tissues, antigen was retrieved in 10 mM citrate buffer, pH 6.0, containing 0.05% Tween-20. The tissues were sequentially treated with 3% hydrogen peroxide and Ultra V block solution. After 1 h incubation at RT with GLS1 antibody (1:250 diluted, Abcam, Cambridge, MA, USA), the slides were washed in Tris-buffered saline including Tween-20 (TBST), incubated with primary antibody enhancer for 10 min, and exposed to horseradish peroxidase-conjugated secondary antibody for 15 min. After re-washing in TBST, the tissue slides were incubated with diaminobenzidine chromogen (Scytek Laboratories Inc, Logan, UT, USA) and counter-stained with Mayer's hematoxylin (Dako Cytomation, Glostrup, Denmark). To evaluate GLS1 expression level, the staining intensity was scored on a scale of 0–3: 0, no staining of cancer cells; 1, weak staining; 2, moderate staining; 3, strong staining. In addition, the percentage of positive cells in tissue microarray core was scored. These two scores were multiplied and the resulting value was used as the expression score.

### Sulforhodamine B assay: cell growth assay

Cells (100 μl) were inoculated into 96-well microtitre plates at plating densities ranging from 5000 to 40 000 cells/well depending on the doubling time of the individual cell line. After cell inoculation, the microtitre plates were incubated for 24 h prior to the addition of the experimental drugs. The drugs were prepared at the appropriate concentrations and 100 μl was added to each well; the plates were then incubated in CO_2_ incubator. The assay was terminated by the addition of cold TCA. The cells were fixed *in situ* by gently adding 50 μl of cold 50% (w/v) TCA (final concentration, 10% TCA) and incubated for 60 min at 4 °C. The supernatant was discarded, and the plates were washed five times with tap water and then air dried. Sulforhodamine B solution (100 μl) at 0.4% (w/v) in 1% acetic acid was added to each well, and the plates were then left for 10 min at room temperature. After staining, the unbound dye was removed by washing five times with 1% acetic acid; the plates were then air dried. The bound stain was subsequently solubilized with 10 mM trizma base, and the absorbance was recorded using an automated plate reader at 515 nm.

### Western blot

Western blots were performed as previously described.^19^ Briefly, the cells were harvested, washed in phosphate-buffered saline (PBS) and lysed in lysis buffer (20 mM Tris-HCl (pH 7.4), 150 mM NaCl, 1% (v/v) Triton X-100, 1 mM EDTA and protease inhibitors). The lysates were analysed using western blot.

### Measurement of ATP and glutamate levels

Total ATP levels were monitored using an ATP Colorimetric/Fluorometric Assay Kit as per the manufacturer's instructions (BioVision, Milpitas, CA, USA). The cells (1 × 10^6^) were lysed in 100 μl of ATP assay buffer and then centrifuged under ice-cold conditions at 15 000 *g* for 2 min to pellet the insoluble materials. The supernatant was collected, and 2–50 μl of this supernatant was added to a 96-well plate, with the final volume topped up to 50 μl/well with ATP assay buffer. ATP reaction mix was made (ATP assay buffer 44 μl, ATP probe 2 μl, ATP converter 2 μl and developer mix 2 μl), and 50 μl of this reaction mix was added to each well containing a test sample. Then, the plate was incubated at room temperature for 30 min in the dark, and the OD was measured at 570 nm using a microplate reader.

Glutamate levels were monitored using a Glutamate Assay Kit as per the manufacturer's instructions (BioVision). The cells (1 × 10^6^) were lysed in 100 μl of assay buffer and then centrifuged under ice-cold conditions at 13 000 *g* for 10 min to pellet the insoluble materials. The supernatant was then collected, and 10–50 μl of this supernatant was added to a 96-well plate; the final volume was topped up to 50 μl/well with assay buffer. Reaction mix was made (assay buffer 90 μl, glutamate developer 8 μl and glutamate enzyme mix 2 μl), and 50 μl of the reaction mix was added to each well containing a test sample. Then, the plate was incubated at 37 °C for 30 min in the dark, and the OD was measured at 450 nm using a microplate reader.

### 2',7'-dichlorofluorescin diacetate cellular ROS detection assay

ROS levels were monitored using a 2',7'-dichlorofluorescin diacetate Cellular ROS Detection Assay Kit as per the manufacturer's instructions (Abcam, Cambridge, UK). The cells were incubated with or without 10 μM BPTES for 48 h. The cells were then collected, washed twice with cold PBS, centrifuged at 1000 rpm for 3 min, stained in culture media with 20 μM 2',7'-dichlorofluorescin diacetate, and incubated for 30 min at 37 °C. The samples were then analysed by FACS flow cytometry (BD Falcon, Franklin Lakes, NJ, USA).

### Glutathione fluorometric assay

Total glutathione levels were monitored using a Glutathione Fluorometric Assay Kit as per the manufacturer's instructions (BioVision). The cells (2 × 10^6^) were lysed in 100 μl of assay buffer, and 60 μl of each homogenate was added to a prechilled tube containing PCA, which was then vortexed for several seconds to achieve a uniform emulsion. The tubes were kept on ice for 5 min and then centrifuged under ice-cold conditions at 13 000 *g* for 2 min to pellet the insoluble materials. The supernatant was collected, and 20 μl of ice-cold 6 N KOH was added to 40 μl of the PCA-preserved samples to precipitate the PCA and to neutralize the samples. The samples were kept on ice for 2 min and then centrifuged at 13 000 *g* at 4 °C. Ten microlitres of the neutralized samples was transferred to a 96-well plate, and the sample wells were topped up to 80 μl with assay buffer. The reducing agent (10 μl) was added to each well containing a test sample, and the wells were thoroughly mixed and left at room temperature for 10 min. Next, 10 μl of the orthophthaldehyde probe was added to the sample wells, which were mixed well and left at room temperature for 40 min. Lastly, Excitation/Emission=340/420 nm was measured using a fluorescence plate reader.

### Lactate measurement

Cells were treated with BPTES (10 μM) for 48 h. Cell culture media was transferred into a conical tube and centrifuged at 1200 rpm for 5 min. The level of lactic acid was measured using a Bioprofile BASIC 4 (Nova, Waltham, MA, USA).

### Measurement of oxygen consumption rate

OCR measurement was performed using the XF96 Extracellular Flux analyser (Seahorse Bioscience, North Billerica, MA, USA). Briefly, cells were plated into XF96 (V3) polystyrene cell culture plates (Seahorse Bioscience, North Billerica). A549 cells were seeded at 20 000 cells/well (XF96 plate) and EKVX cells were seeded at 10 000 cells/well (XF96 plate), respectively. The cells were incubated for 24 h in a humidified 37 °C incubator with 5% CO_2_. Prior to performing an assay, the growth medium in the wells is allowed to achieve a minimum dilution of 1 : 1000. One hundred seventy-five microliters of the assay medium was added to cells for an extracellular flux assay. While sensor cartridges were calibrated, cell plates were incubated in a 37 °C/non-CO_2_ incubator for 60 min prior to the start of an assay. All experiments were performed at 37 °C. Each measurement cycle consisted of a mixing time of 2 min and data acquisition period time for 4 min. OCR data points refer to the average rates during the measurement cycles. All compounds were prepared at appropriate concentrations in desired assay medium and adjusted to pH 7.4. A volume of 25 μl of compound was added to each injection port. In a typical experiment, three baseline measurements were taken prior to the addition of any compound, and three response measurements were taken after the addition of each compound. OCR was reported as absolute rates (pmoles/min).

### Cell cycle assay

The cells were incubated with or without 10 μM BPTES, 10 μM 5-FU, or both for 48 h. The cells were then collected, washed twice with PBS, centrifuged at 1500 rpm for 5 min and fixed using EtOH over 2 h at 4 °C. They were then centrifuged at 2000 rpm for 10 min, stained with PI+RNase solution for 30 min in the dark, washed with cold PBS, and then analysed by FACS flow cytometry (BD Falcon, Bedford, MA, USA).

### FITC annexin V apoptosis detection

The cells were incubated with or without 10 μM BPTES, 10 μM 5-FU, or both for 48 h. The cells were then collected, washed with cold PBS, centrifuged at 1400 rpm for 3 min, and then resuspended in 1X binding buffer at a concentration of 1 × 10^6^ cells/ml. The solution (100 μl) was transferred (1 × 10^5^) to a 5-ml culture tube, and 5 μl each of annexin V-FITC and PI were added. The cells were gently vortexed and incubated for 15 min at room temperature in the dark. Then, 400 μl of 1X binding buffer was added to each tube, and the samples were analysed by FACS flow cytometry (BD Falcon, Bedford, MA, USA).

### Relative quantitation of metabolites of energy metabolism using liquid chromatography-tandem mass spectrometry (LC-MS/MS)

Standard metabolites and internal standard were purchased from Sigma-Aldrich (St. Louis, MI, USA). All solvents including water were purchased from JT Baker. Cell number would be ~1 million. Cells were harvested using 1.4 ml of cold methanol/H_2_O (80/20, v/v) after sequential wash with PBS and H_2_O. Then cells were lysed by vigorous vortexing and 100 μl of 5 μM of internal standard (13C5-Glutamine) was added. Metabolites were extracted from aqueous phase by liquid-liquid extraction after adding chloroform. The aqueous phase was dried using vacuum centrifuge, and the sample was reconstituted with 50 μl of 50% methanol prior to LC-MS/MS analysis.

Metabolites in energy metabolism were analysed with LC-MS/MS equipped with 1290 HPLC (Agilent, Santa Clara, CA, USA), Qtrap 5500 (ABSciex, Concord, Ontario, Canada), and a reverse phase column (Synergi fusion RP 50 × 2 mm). 3 μl was injected into the LC-MS/MS system and ionized with turbo spray ionization source. 5 mM ammonium acetate in H_2_O and 5 mM ammonium acetate in methanol were used as mobile phase A and B, respectively. The separation gradient was as follows: hold at 0% B for 5 min, 0–90% B for 2 min, hold at 90% for 8 min, 90–0% B for 1 min, then hold at 0% B for 9 min. Liquid chromatography flow was 70 μl/min except 140 μl/min between 7 and 15 min, and column temperature was kept at 23 °C. Multiple reaction monitoring was used in negative ion mode, and the extracted ion chromatogram corresponding to the specific transition for each metabolite was used for quantitation. Multiple reaction monitoring transitions of each metabolite are shown in Supplementary Table 1. Area under the curve of each extracted ion chromatogram was normalized to that of extracted ion chromatogram of internal standard. Peak area ratio of each metabolite to internal standard was normalized using protein amount in a sample, then was used for relative comparison. Data analysis was performed using Analyst 1.5.2 software.

### *In situ* cell death detection (TUNEL assay)

The samples were incubated with or without 10 μM BPTES, 10 μM 5-FU, or both for 48 h. The samples were washed with cold PBS for 3 min, and air-dried cell samples were fixed with a freshly prepared fixation solution (4% paraformaldehyde in PBS, pH 7.4) for 1 h at room temperature. The samples were washed with PBS for 5 min and then incubated in permeabilization solution (0.1% Triton X-100 in 0.1% sodium citrate) for 2 min on ice. Then, the samples were washed with cold PBS for 5 min, and 50 μl of TUNEL reaction mixture was added to each sample after drying the area around the sample. The samples were incubated for 1 h at 37 °C in the dark, washed with cold PBS for 10 min and then analysed under a fluorescence microscope.

### Preclinical xenograft tumour models

Balb/c-nu mice (Central Lab Animal, Seoul, Korea) were aged between 6 and 8 weeks before tumour induction. This study was reviewed and approved by the Institutional Animal Care and Use Committee of the National Cancer Center Research Institute (NCCRI; protocols: NCC-15-289). NCCRI is an Association for Assessment and Accreditation of Laboratory Animal Care International-accredited facility and abides by the guidelines of the Institute of Laboratory Animal Resources.

A549-luc-C8 cells (5.0 × 10^6^) were subcutaneously inoculated using a 1-ml syringe. After 2 weeks, the mice were divided into four groups: a control group treated with vehicle (polyethylene glycol 50%, PBS 40%, DMSO 10%), BPTES, 5-FU, or both BPTES and 5-FU (BPTES 10 mg/kg/200 μl, 5-FU 20 mg/kg/200 μl, each group 10 heads).

### Statistical analysis

Statistical analysis was performed using the Student's *t*-test, except in the comparison of GLS1 expression levels between normal lung and NSCLC tissues, in which the Mann–Whitney *U* test was used.

## Supplementary Material

Supplementary Figures1-4, Table 1

## References

[bib1] Gao P, Tchernyshyov I, Chang T-C, Lee Y-S, Kita K, Ochi T et al. c-Myc suppression of miR-23a/b enhances mitochondrial glutaminase expression and glutamine metabolism. Nature 2009; 458: 762–765. 19219026 10.1038/nature07823 PMC2729443

[bib2] Le A, Lane AN, Hamaker M, Bose S, Gouw A, Barbi J et al. Glucose-independent glutamine metabolism via TCA cycling for proliferation and survival in B cells. Cell Metab 2012; 15: 110–121. 22225880 10.1016/j.cmet.2011.12.009 PMC3345194

[bib3] Yuneva MO, Fan TW, Allen TD, Higashi RM, Ferraris DV, Tsukamoto T et al. The metabolic profile of tumors depends on both the responsible genetic lesion and tissue type. Cell Metab 2012; 15: 157–170. 22326218 10.1016/j.cmet.2011.12.015 PMC3282107

[bib4] van den Heuvel APJ, Jing J, Wooster RF, Bachman KE. Analysis of glutamine dependency in non-small cell lung cancer: GLS1 splice variant GAC is essential for cancer cell growth. Cancer Biol Ther 2012; 13: 1185–1194. 22892846 10.4161/cbt.21348 PMC3469476

[bib5] Xiang Y, Stine ZE, Xia J, Lu Y, O'Connor RS, Altman BJ et al. Targeted inhibition of tumor-specific glutaminase diminishes cell-autonomous tumorigenesis. J Clin Invest 2015; 125: 2293–2306. 25915584 10.1172/JCI75836 PMC4497742

[bib6] Weinberg F, Hamanaka R, Wheaton WW, Weinberg S, Joseph J, Lopez M et al. Mitochondrial metabolism and ROS generation are essential for Kras-mediated tumorigenicity. Proc Natl Acad Sci 2010; 107: 8788–8793. 20421486 10.1073/pnas.1003428107 PMC2889315

[bib7] DeBerardinis RJ, Mancuso A, Daikhin E, Nissim I, Yudkoff M, Wehrli S et al. Beyond aerobic glycolysis: transformed cells can engage in glutamine metabolism that exceeds the requirement for protein and nucleotide synthesis. Proc Natl Acad Sci 2007; 104: 19345–19350. 18032601 10.1073/pnas.0709747104 PMC2148292

[bib8] Kang JH, Lee SH, Lee JS, Nam B, Seong TW, Son J et al. Aldehyde dehydrogenase inhibition combined with phenformin treatment reversed NSCLC through ATP depletion. Oncotarget 2016 (doi:10.18632/oncotarget.10354; e-pub ahead of print). 10.18632/oncotarget.10354 PMC5226516 27384481

[bib9] Zu XL, Guppy M. Cancer metabolism: facts, fantasy, and fiction. Biochem Biophys Res Commun 2004; 313: 459–465. 14697210 10.1016/j.bbrc.2003.11.136

[bib10] Moreno-Sanchez R, Marin-Hernandez A, Saavedra E, Pardo JP, Ralph SJ, Rodriguez-Enriquez S. Who controls the ATP supply in cancer cells? Biochemistry lessons to understand cancer energy metabolism. Int J Biochem Cell Biol 2014; 50: 10–23. 24513530 10.1016/j.biocel.2014.01.025

[bib11] Lee JS, Kang JH, Lee SH, Lee CH, Son J, Kim SY. Glutaminase 1 inhibition reduces thymidine synthesis in NSCLC. Biochem Biophys Res Commun 2016; 477: 374–382. 27338638 10.1016/j.bbrc.2016.06.095

[bib12] Győrffy B, Surowiak P, Budczies J, Lánczky A. Online survival analysis software to assess the prognostic value of biomarkers using transcriptomic data in non-small-cell lung cancer. PloS One 2013; 8: e82241. 24367507 10.1371/journal.pone.0082241 PMC3867325

[bib13] Sweet S, Singh G. Accumulation of human promyelocytic leukemia (HL-60) cells at two energetic cell cycle checkpoints. Cancer Res 1995; 55: 5164–5167. 7585566

[bib14] Sweet S, Singh G. Changes in mitochondrial mass, membrane potential, and cellular adenosine triphosphate content during the cell cycle of human leukemic (HL-60) cells. J Cell Physiol 1999; 180: 91–96. 10362021 10.1002/(SICI)1097-4652(199907)180:1<91::AID-JCP10>3.0.CO;2-6

[bib15] Dang CV, Le A, Gao P. MYC-induced cancer cell energy metabolism and therapeutic opportunities. Clin Cancer Res 2009; 15: 6479–6483. 19861459 10.1158/1078-0432.CCR-09-0889 PMC2783410

[bib16] DeBerardinis RJ, Cheng T. Q's next: the diverse functions of glutamine in metabolism, cell biology and cancer. Oncogene 2010; 29: 313–324. 19881548 10.1038/onc.2009.358 PMC2809806

[bib17] Greenhouse WV, Lehninger AL. Occurrence of the malate-aspartate shuttle in various tumor types. Cancer Res 1976; 36: 1392–1396. 177206

[bib18] Li J, Csibi A, Yang S, Hoffman GR, Li C, Zhang E et al. Synthetic lethality of combined glutaminase and Hsp90 inhibition in mTORC1-driven tumor cells. Proc Natl Acad Sci 2015; 112: E21–E29. 25524627 10.1073/pnas.1417015112 PMC4291663

[bib19] Ku BM, Kim DS, Kim KH, Yoo BC, Kim SH, Gong YD et al. Transglutaminase 2 inhibition found to induce p53 mediated apoptosis in renal cell carcinoma. FASEB J 2013; 27: 3487–3495. 23704086 10.1096/fj.12-224220

