# Supplementary material for: Dual targeting of glutaminase 1 and thymidylate synthase elicits death synergistically in NSCLC
Source: Cell Death Dis. 2016 Dec 8;7(12):e2511–. doi: 10.1038/cddis.2016.404 (PMC5261012; doi:10.1038/cddis.2016.404)
Supplement: Supplementary Figures1-4, Table 1 [file cddis2016404x1.ppt]

## Slide 1
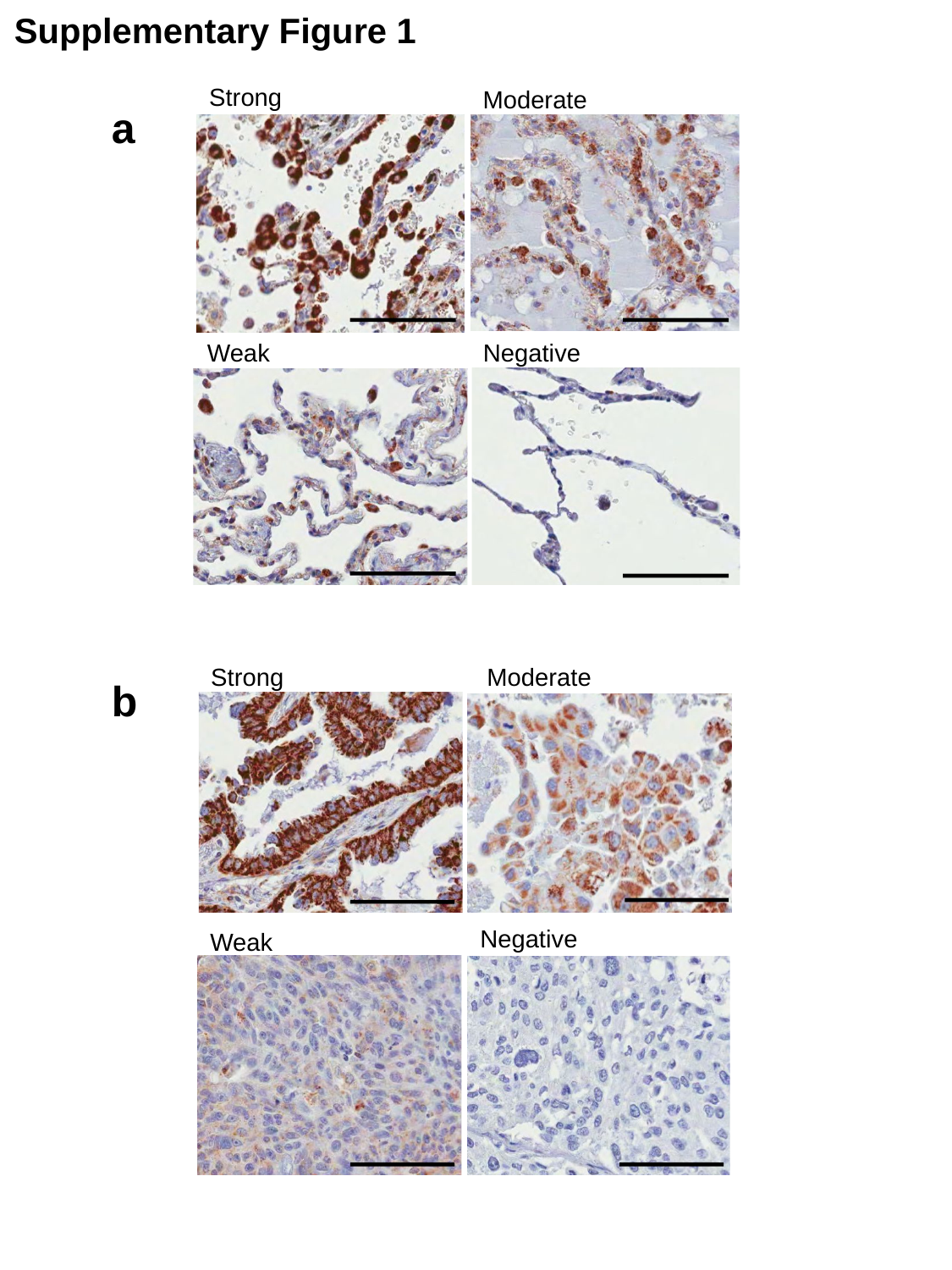

Supplementary Figure 1
Strong
Moderate
a
Weak
Negative
Strong
Moderate
b
Negative
Weak

## Slide 2
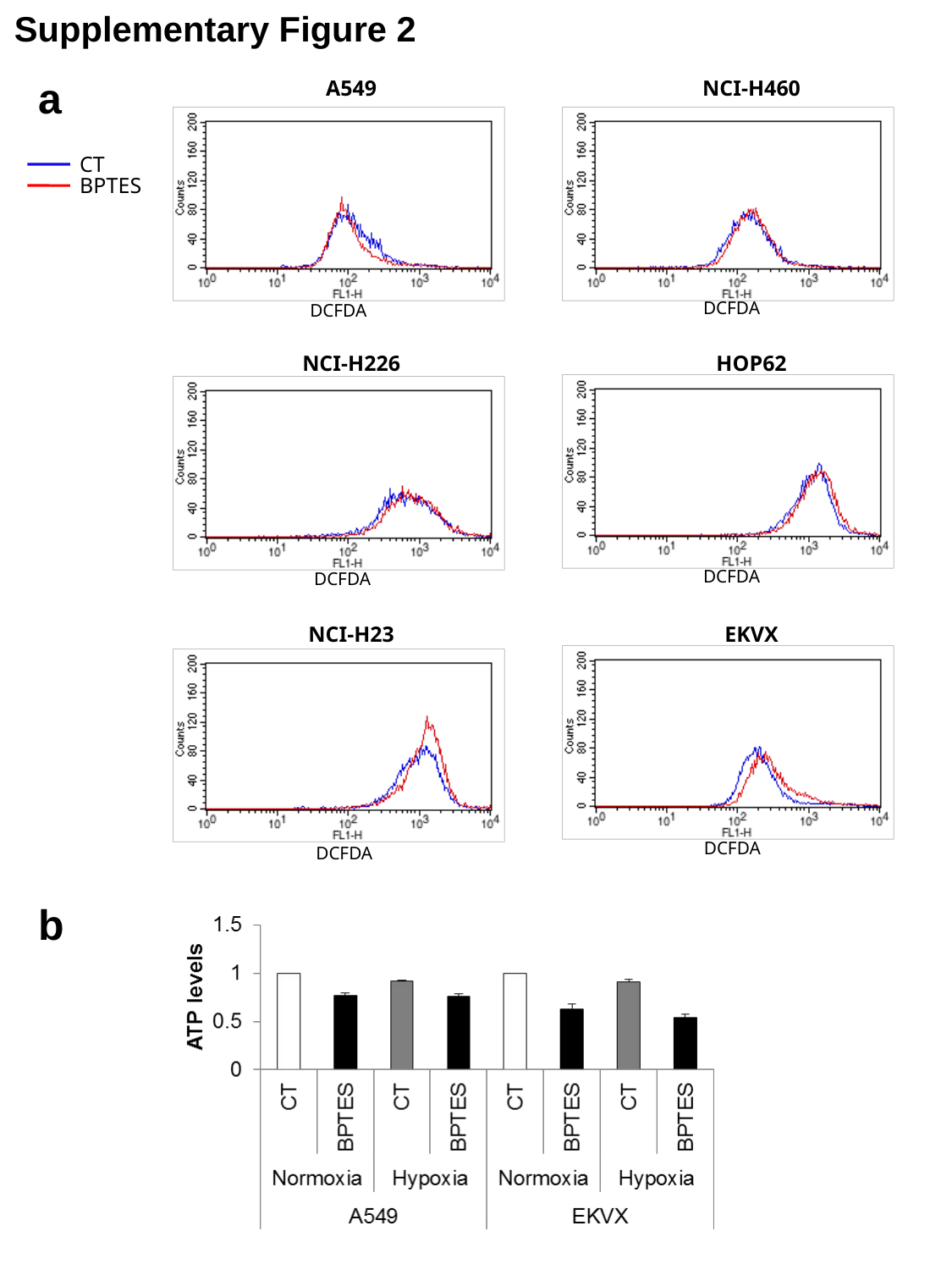

Supplementary Figure 2
a
A549
NCI-H460
CT
BPTES
DCFDA
DCFDA
NCI-H226
HOP62
DCFDA
DCFDA
NCI-H23
EKVX
DCFDA
DCFDA
b

## Slide 3
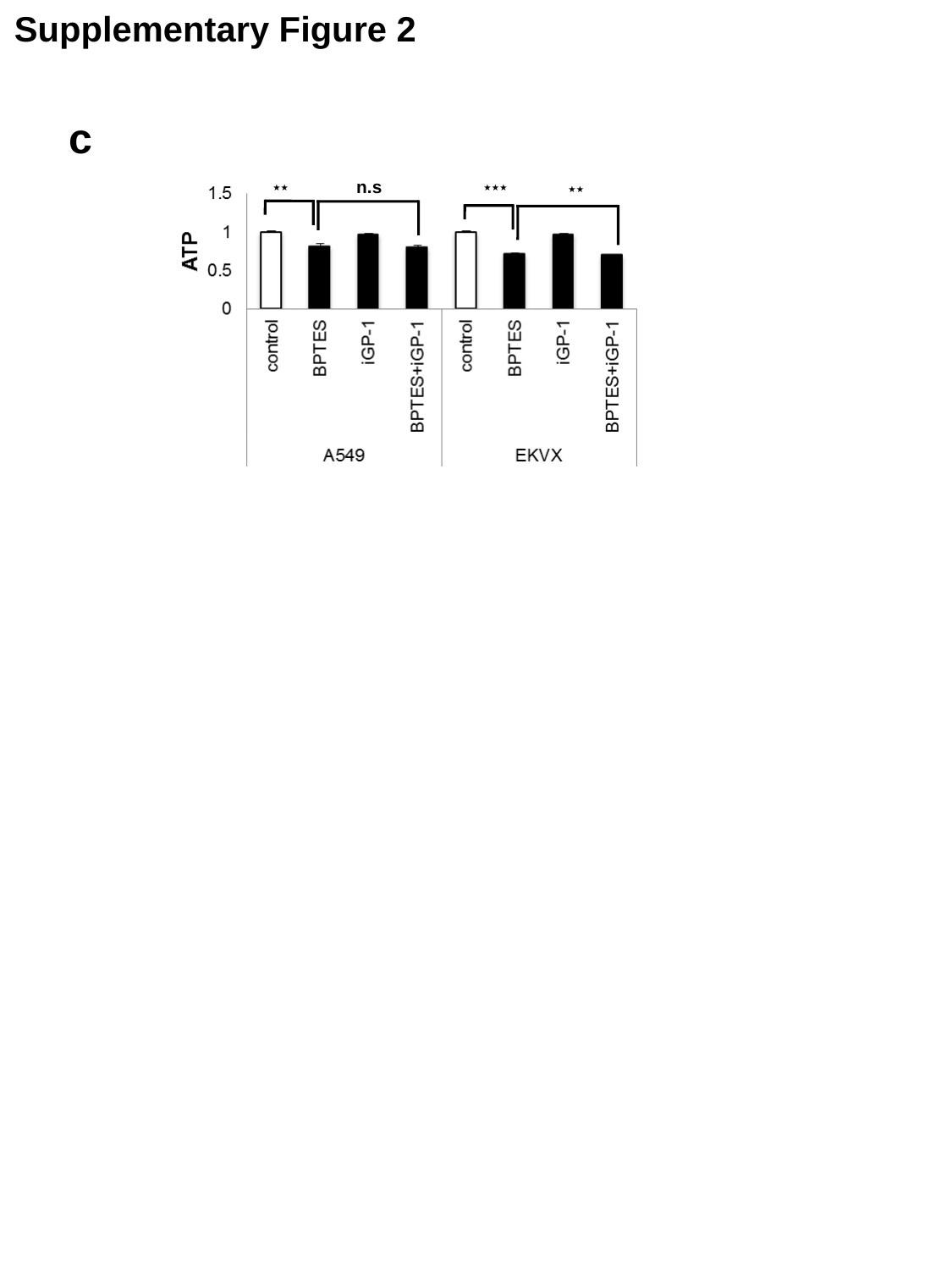

Supplementary Figure 2
c
n.s
★★
★★★
★★

## Slide 4
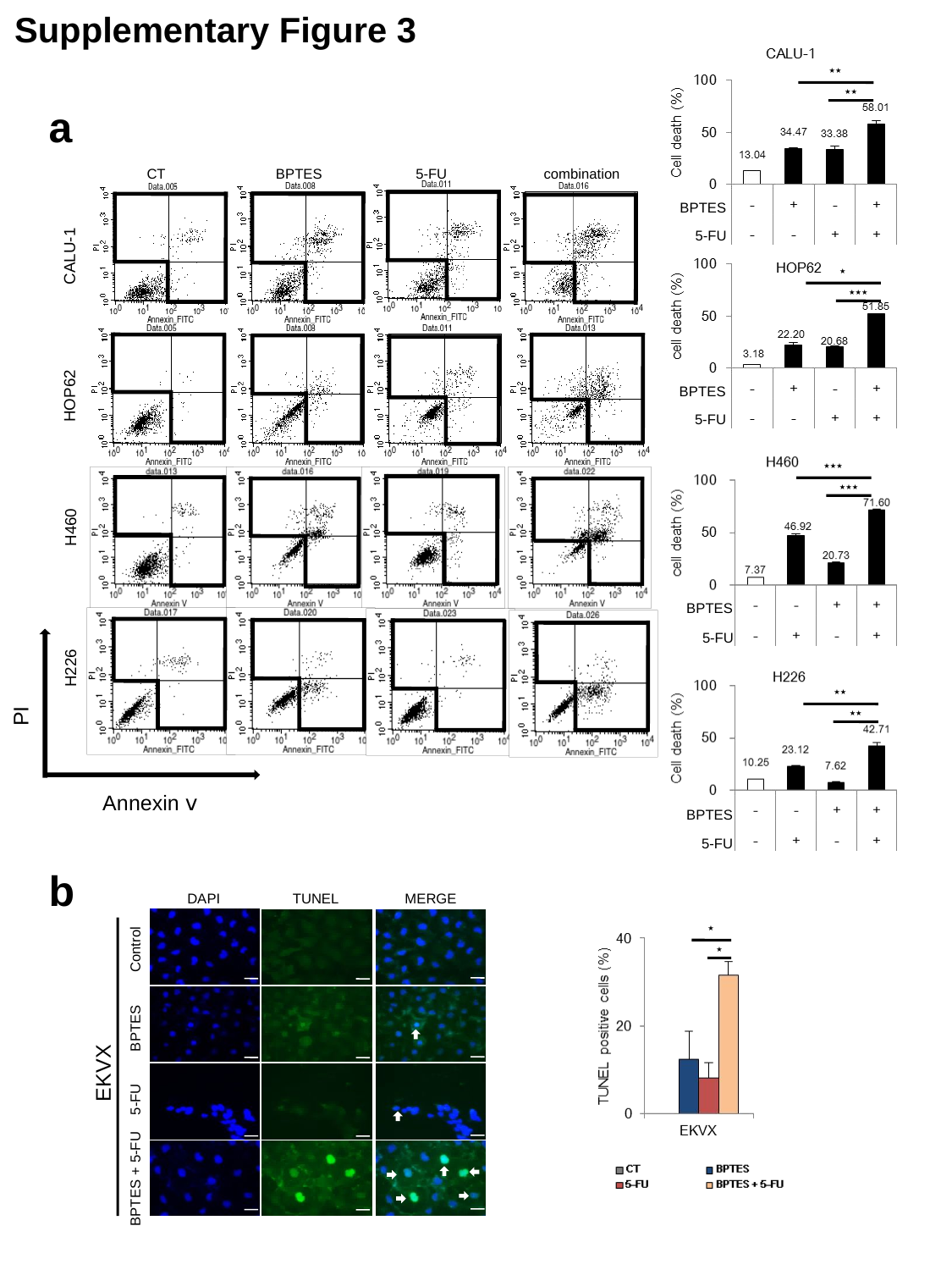

Supplementary Figure 3
BPTES
5-FU
★★
★★
a
CT
BPTES
5-FU
combination
BPTES
5-FU
CALU-1
★
★★★
HOP62
BPTES
5-FU
★★★
★★★
H460
BPTES
5-FU
H226
★★
★★
PI
.
.
Annexin ⅴ
b
DAPI
TUNEL
MERGE
Control
★
★
BPTES
EKVX
5-FU
BPTES + 5-FU

## Slide 5
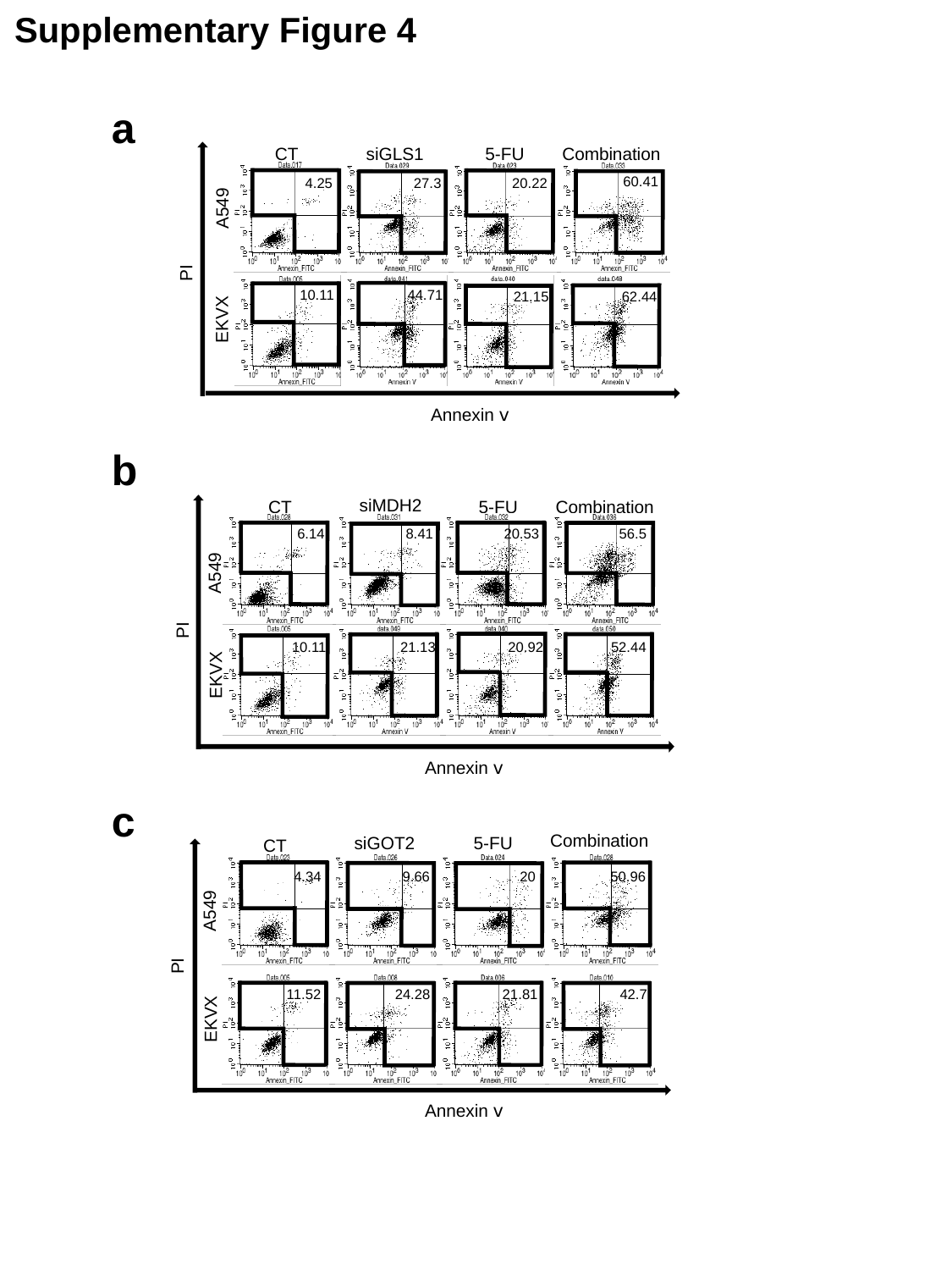

Supplementary Figure 4
a
A549
CT
siGLS1
5-FU
Combination
60.41
4.25
27.3
20.22
PI
.
EKVX
10.11
44.71
21.15
62.44
.
Annexin ⅴ
b
A549
siMDH2
CT
5-FU
Combination
6.14
8.41
20.53
56.5
PI
.
10.11
21.13
20.92
52.44
EKVX
.
Annexin ⅴ
c
A549
Combination
siGOT2
5-FU
CT
4.34
9.66
20
50.96
PI
.
11.52
24.28
21.81
42.7
EKVX
.
Annexin ⅴ

## Slide 6
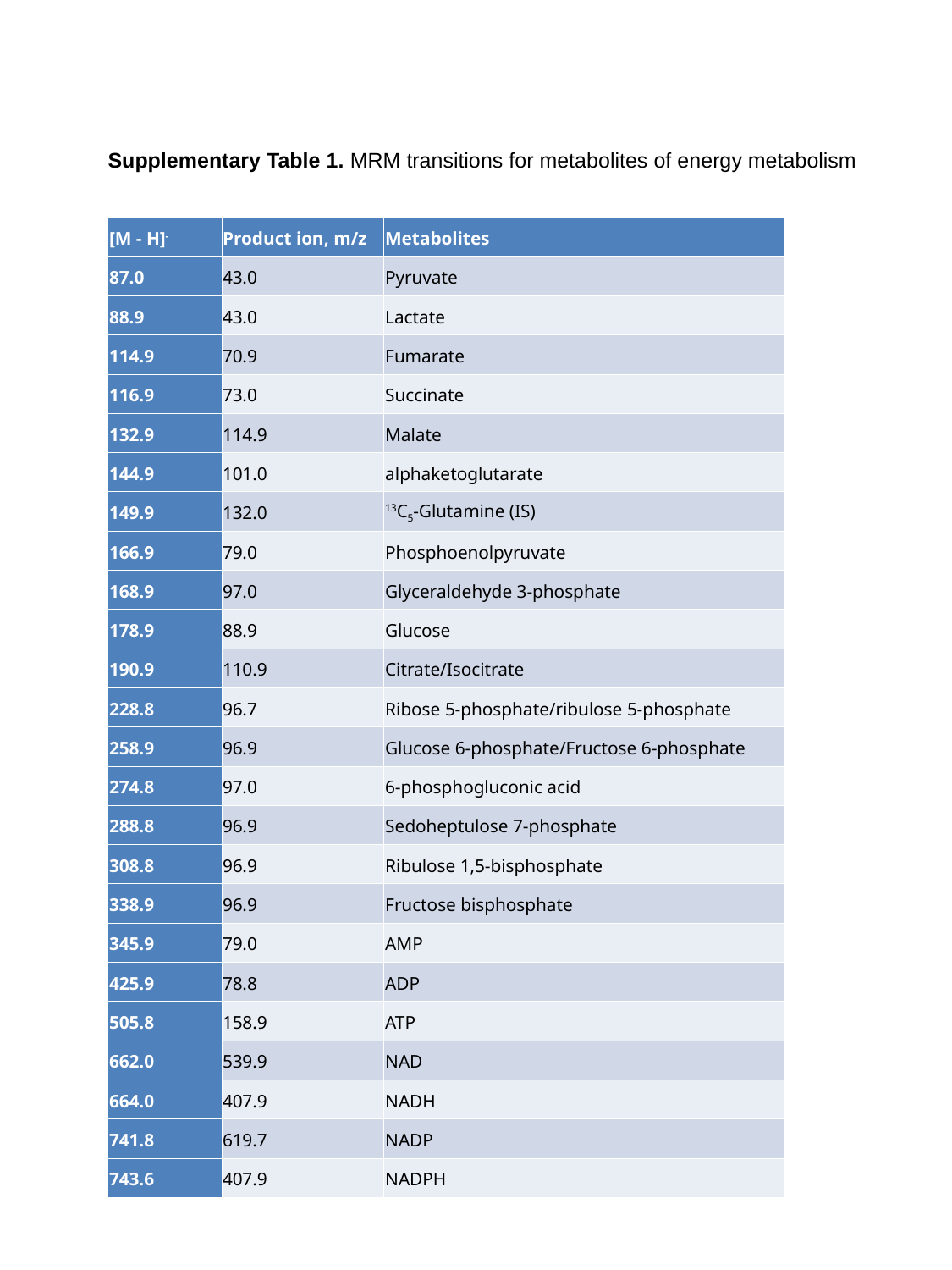

Supplementary Table 1. MRM transitions for metabolites of energy metabolism
| [M - H]- | Product ion, m/z | Metabolites |
| --- | --- | --- |
| 87.0 | 43.0 | Pyruvate |
| 88.9 | 43.0 | Lactate |
| 114.9 | 70.9 | Fumarate |
| 116.9 | 73.0 | Succinate |
| 132.9 | 114.9 | Malate |
| 144.9 | 101.0 | alphaketoglutarate |
| 149.9 | 132.0 | 13C5-Glutamine (IS) |
| 166.9 | 79.0 | Phosphoenolpyruvate |
| 168.9 | 97.0 | Glyceraldehyde 3-phosphate |
| 178.9 | 88.9 | Glucose |
| 190.9 | 110.9 | Citrate/Isocitrate |
| 228.8 | 96.7 | Ribose 5-phosphate/ribulose 5-phosphate |
| 258.9 | 96.9 | Glucose 6-phosphate/Fructose 6-phosphate |
| 274.8 | 97.0 | 6-phosphogluconic acid |
| 288.8 | 96.9 | Sedoheptulose 7-phosphate |
| 308.8 | 96.9 | Ribulose 1,5-bisphosphate |
| 338.9 | 96.9 | Fructose bisphosphate |
| 345.9 | 79.0 | AMP |
| 425.9 | 78.8 | ADP |
| 505.8 | 158.9 | ATP |
| 662.0 | 539.9 | NAD |
| 664.0 | 407.9 | NADH |
| 741.8 | 619.7 | NADP |
| 743.6 | 407.9 | NADPH |
